# Differential effects of interleukin-17A and 17F on cell interactions between immune cells and stromal cells from synovium or skin

**DOI:** 10.1038/s41598-023-45653-8

**Published:** 2023-11-06

**Authors:** Issam Tout, Mélissa Noack, Pierre Miossec

**Affiliations:** 1 Immunogenomics and Inflammation Research Unit, Hospices Civils de Lyon, Edouard Herriot Hospital, 5 Place d’Arsonval, 69003 Lyon, France; 2 Department of Clinical Immunology and Rheumatology, Edouard Herriot Hospital, 5 Place d’Arsonval, 69437 Lyon, France

**Keywords:** Immunology, Rheumatology

## Abstract

We compared the contribution of IL-17A and IL-17F in co-culture systems mimicking cell interactions as found in inflamed synovium and skin. Synoviocytes or skin fibroblasts were co-cultured with activated PBMC, with IL-17A, IL-17 A/F, IL-17F, IL-23, anti-IL-17A, anti-IL-17A/F or anti-IL-17F antibodies. IL-17A, IL-17F, IL-6 and IL-10 production was measured at 48 h. mRNA expression of receptor subunits for IL-23, IL-12 and IL-17 was assessed at 24 h. Both cell activation and interactions were needed for a high IL-17A secretion while IL-17F was stimulated by PHA activation alone and further increased in co-cultures. IL-17F levels were higher than IL-17A in both co-cultures (p < 0.05). IL-17F addition decreased IL-17A secretion (p < 0.05) but IL-17A addition had no effect on IL-17F secretion. Interestingly, IL-17A and IL-17F upregulated IL-17RA and IL-17RC mRNA expression in PBMC/skin fibroblast co-cultures (p < 0.05) while only IL-17F exerted this effect in synoviocytes (p < 0.05). Monocyte exclusion in both co-cultures increased IL-17A and IL-17F (twofold, p < 0.05) while decreasing IL-10 and IL-6 secretion (twofold, p < 0.05). IL-17A and F had differential effects on their receptor expression with a higher sensitivity for skin fibroblasts highlighting the differential contribution of IL-17A and F in joint vs. skin diseases.

## Introduction

During chronic inflammation, as in inflammatory arthritis or psoriasis (Pso), immune cells migrate to inflammatory sites, where they interact with local stromal cells, synoviocytes and skin fibroblasts, respectively. This immune infiltrate includes helper 17 (Th17) CD4 T cells and other IL-17-producing cells^1, 2^. Such accumulation is found in sections of psoriatic skin^3^ and synovial tissue in rheumatoid and psoriatic arthritis (RA, PsA)^4^. In RA, IL-17A induces changes in the synovium that lead to synovitis and chronic inflammation^5^. These interactions induce the survival of pathogenic immune and of stromal cells and their proliferation through acquired apoptosis defects. This results in the production of pathogenic autoantibodies and cytokines^6, 7^.

Members of the IL-17 family (IL-17A to IL-17F) mediate their biological functions through a family of IL-17 receptors (IL-17RA to IL-17RE). IL-17A, the first member of the family has been much more studied than IL-17F, the most similar (55% sequence analogy) IL-17 family member. These are homodimers and there is also an IL-17A-IL-17F (IL-17A/F) heterodimer that has been poorly studied^8^. All these members act through binding to IL-17RA and IL-17RC receptor complex^9^.

The crucial role of IL-17 in Pso and PsA as well as IL-17 involvement in RA pathogenesis has resulted in many biologic therapies targeting IL-17 members. These involve the use of antibodies targeting IL-17A, such as secukinumab and ixekizumab^10^ or IL-17RA with brodalumab, and more recently, both IL-17A and F with bimekizumab. Different outcomes have been seen, for instance, an impressive efficacy in Pso^11^ but limited and conflicting results in RA^12^.

Using an in vitro co-culture model^13^, this study compares the contribution of these IL-17 family members and their inhibition during interactions between immune cells and synoviocytes versus skin fibroblasts. We have analyzed the effects of exogenous cytokines (IL-17A, IL-17F, IL-17A/F and IL-23) or blocking antibodies against the same IL-17 family members (anti-IL-17A, anti-IL-17F and anti-IL-17A/F antibodies) on cytokine production and cytokine receptor gene expression. Since monocytes differentiate into dendritic cells and macrophages, which are main sources of IL-23^13, 14^, monocyte contribution was also investigated.

## Results

### Differences in IL-17A versus IL-17F secretion during cell interactions

Synoviocytes and lesional skin fibroblasts were co-cultured in the presence of PHA-activated PBMC. As previously described^15^, this model mimics the cell interactions seen at the inflammatory site between stromal and immune cells, leading to massive pro-inflammatory cytokine production. This includes IL-17, mainly secreted by Th17 cells and contributing to inflammation exacerbation. This effect is mainly due to cell–cell contact and not to soluble factors, as it is inhibited by using a Transwell system for both synoviocytes and skin fibroblasts^13, 14^. The system was further validated by showing similar results, with an autologous system, with PBMC and synoviocytes or skin fibroblasts from the same patients^13^. The use of the same PBMC in contact with different stromal cells allows the study of stromal cell heterogeneity.

**Figure 1:**
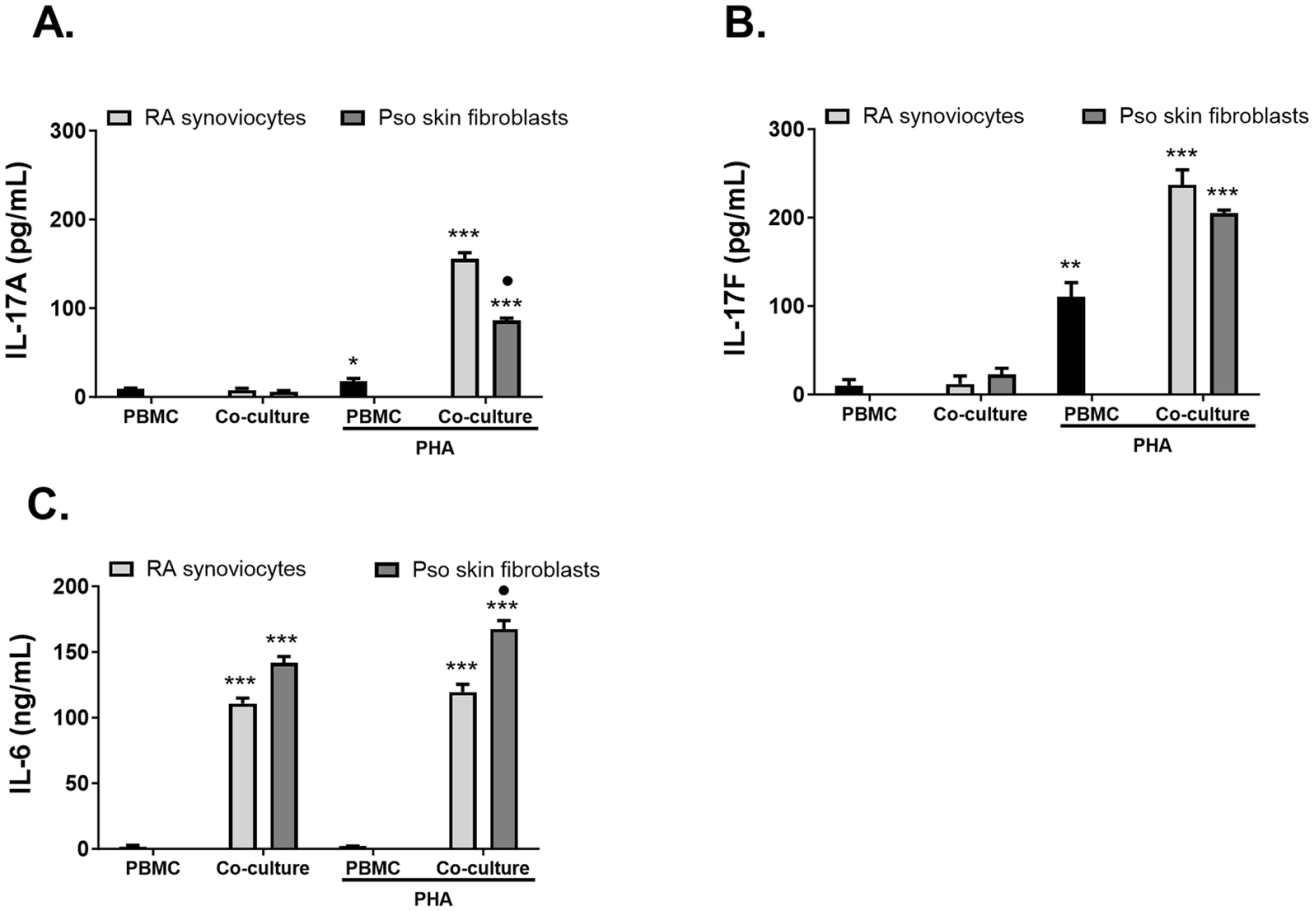
Effects of cell interactions between PBMC and stromal cells from different origins on cytokine production. PBMC were cultured alone or in co-culture with synoviocytes or with lesional skin fibroblasts at a 5:1 ratio for 48 h, in the presence or absence of PHA (5 μg/mL). Production of IL-17A (**A**), IL-17F (**B**) and IL-6 (**C**) in cell supernatants was measured by ELISA. “*” compares the effect of the PHA activation and co-culture vs. the non-stimulated PBMC condition. *p < 0.05; **p < 0.01; ***p < 0.005. “•” Compares the effect of interaction with stromal cells from different origin on cytokine production (synoviocyte vs. skin fibroblast) within the PHA-activated co-culture. ^ns^p > 0.05; ^•^p < 0.05. Results are presented as mean ± SD, n = 4 experiments for PBMC and co-cultures.

In both co-culture models, these cell–cell interactions lead to massive cytokine production, compared to PBMC alone (Fig. 1). A high IL-17A secretion was only obtained in co-cultures with PHA-activated PBMC in both models (synoviocytes: 155.8 vs. 9.1 pg/mL, respectively; skin fibroblasts: 86.0 vs. 11.5 pg/mL, respectively, Fig. 1A). PBMC alone produced low amounts of IL-17A while activated PBMC alone produced slightly higher IL-17A in comparison (9.1 vs. 17.9 pg/mL, respectively, Fig. 1A). IL-17A levels were notably higher in PBMC/synoviocyte compared to PBMC/skin fibroblast co-cultures (155.8 vs. 86.0 pg/mL respectively, Fig. 1A). These results demonstrated that the combination of PHA activation and cell–cell contact was required to obtain a high IL-17A secretion and confirmed previous results obtained^13, 14^.

IL-17F secretion was already stimulated by PHA activation in PBMC alone and further increased by co-culture (synoviocytes: 110.1 vs. 236.9 pg/mL, respectively; skin fibroblasts: 110.1 vs. 204.7 pg/mL, respectively, Fig. 1B). IL-17F production was not significantly different between synoviocyte and skin fibroblast co-cultures (236.9 vs. 204.7 pg/mL, respectively, Fig. 1B). In the unstimulated PBMC alone condition and even when co-cultured with stromal cells, IL-17F levels were extremely low (< 20 pg/mL) showing that the PHA activation is sufficient for IL-17F secretion in PBMC alone and is further increased by the co-culture. This is not the case for IL-17A that needed both PHA activation and co-culture to be actively secreted (Fig. 1A).

IL-6 plays a pivotal role in the pathophysiology of RA. It is found in abundance in the synovial fluid and serum of patients with RA and its levels correlate with disease activity and joint destruction. Moreover, IL-6 mediates inflammation, boosts Th17 differentiation and activates synoviocytes^16^. In the control condition without PHA, cell contact alone between synoviocytes or skin fibroblasts and PBMC was sufficient to induce a massive IL-6 production, a key cytokine for Th17 cell induction (110.6 and 141.7 ng/mL, respectively, Fig. 1C). PBMC alone secreted negligible amounts of IL-6 whether activated or not (Fig. 1C). Moreover, non-activated and PHA-activated PBMC/stromal cell co-cultures exhibited similar IL-6 secretion in both models (Fig. 1C). Interestingly, IL-6 production was higher in skin fibroblast compared to synoviocyte co-cultures (167.4 vs. 119.4 ng/mL, respectively, Fig. 1C).

### Effects of exogenous IL-17 cytokines or their inhibition on cytokine secretion

To assess the effect of exogenous IL-17 members and their inhibition on pro-inflammatory cytokine secretion in co-cultures, cells were treated with exogenous cytokines: IL-17A, IL-17A/F, IL-17F and IL-23 and the following monoclonal antibodies: anti-IL-17A, anti-IL-17A/F and anti-IL-17F. After 48 h, supernatants were recovered and IL-17A, IL-17F and IL-6 secretion was measured by ELISA.

**Figure 2:**
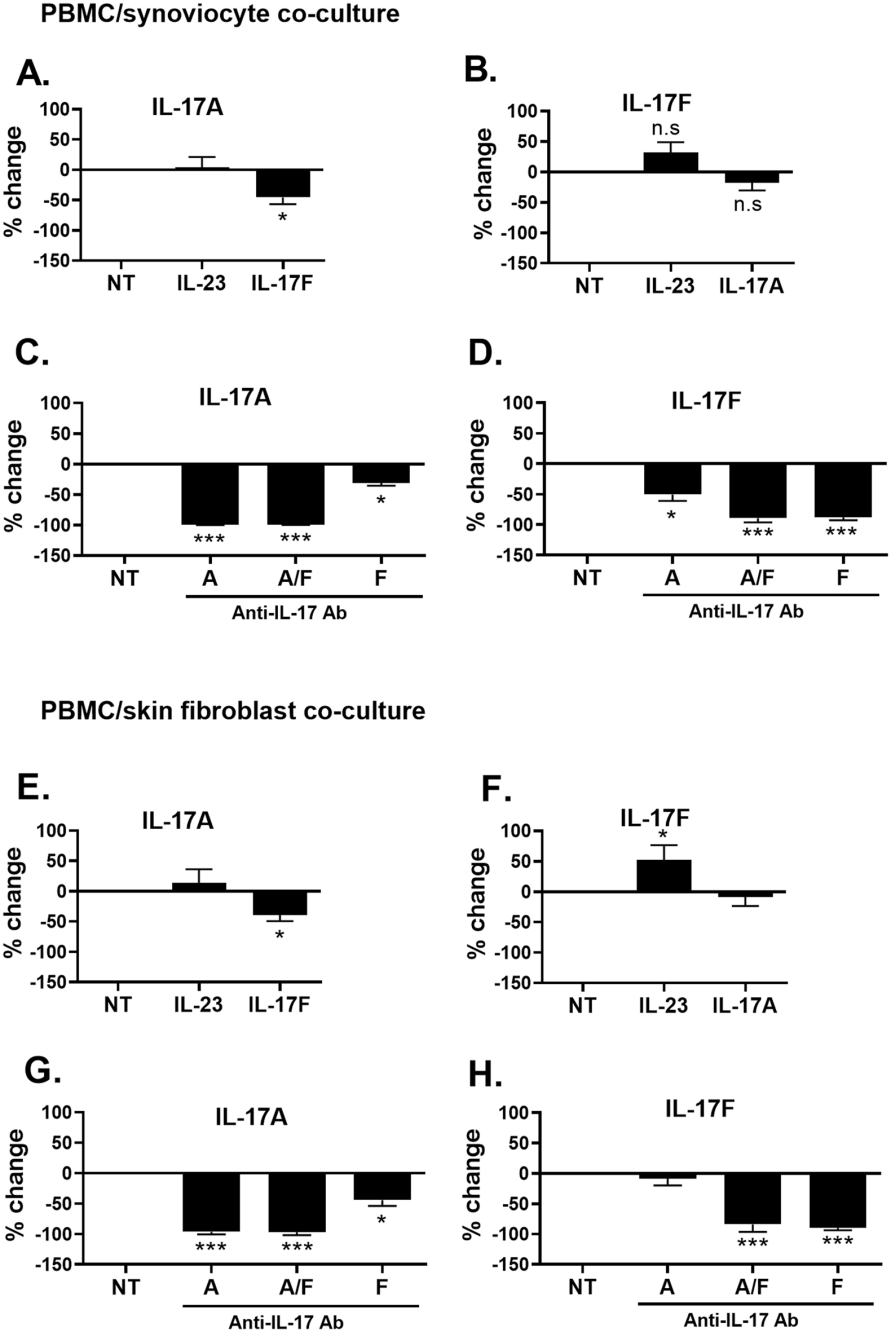
Effects of exogenous cytokines and anti-IL-17 antibodies on IL-17A and IL-17F production in co-cultures between PBMC and stromal cells from different origins. PHA-activated PBMC were treated or not with exogenous cytokines (IL-23, IL-17A, IL-17A/F and IL-17F) or antibodies (anti-IL-17A, anti-IL-17A/F and anti-IL-17F) and co-cultured with synoviocytes (**A–D**) or with skin fibroblasts (**E–H**) at a 5:1 ratio for 48 h. Production of IL-17A and IL-17F was measured by ELISA. *p < 0.05; **p < 0.01; ***p < 0.005. Cytokine production was expressed as a percentage of change compared to the control condition, which is the non-treated condition (NT) and used as 100% control. Results are presented as mean ± SD, n = 5 to 7 experiments.

Results are presented in Fig. 2 and in Supplemental Figs. S1 and S2. Cytokine production was expressed as a percentage of change compared to the control condition, which is the non-treated condition (NT) and used as 100% control. Cytokine secretion was either upregulated (positive values) or downregulated (negative values) compared to the control. IL-17A production was decreased by IL-17F addition, by nearly twofold, in both synoviocyte or skin fibroblast co-cultures (Fig. 2A,E).

Anti-IL-17A and anti-IL-17A/F antibodies strongly decreased IL-17A production in both PBMC/synoviocyte (Fig. 2C) and PBMC/skin fibroblast co-cultures (Fig. 2G). Remarkably, anti-IL-17F decreased IL-17A levels to a lesser extent than anti-IL-17A and anti-IL-17A/F (twofold decrease, Fig. 2G) in PBMC/skin fibroblast co-cultures, and a weaker but still significant downregulation was seen in PBMC/synoviocyte co-cultures (− 31.3%, p = 0.0375, Fig. 2C). IL-17A concentration decrease by anti-IL-17A and anti-IL-17A/F in both co-cultures (reaching nearly 1–2 pg/mL) are shown in Supplementary Fig. S1A,C.

IL-17A addition did not seem to affect IL-17F levels in both synoviocyte and skin fibroblast co-cultures despite a noted tendency to decrease it in synoviocytes, but without reaching statistical significance (− 17.9% and − 8.5%, respectively, p = 0.0625 and p = 0.275, Fig. 2B,F). This shows that the interplay between IL-17A and IL-17F is not reciprocal as IL-17F addition decreased IL-17A secretion with IL-17A addition having no effect on IL-17F production. Anti-IL-17A/F and anti-IL-17F antibodies strongly decreased IL-17F levels in both PBMC/synoviocyte (Fig. 2D, Fig. S1B) and skin fibroblast co-cultures (Fig. 2H, Fig. S1D) while anti-IL-17A decreased IL-17F levels only in PBMC/synoviocyte co-cultures (− 50.5%, p = 0.0236, Fig. 2D, Fig. S1B) with no significant effect on IL-17F in PBMC/skin fibroblast (Fig. 2H, Fig. S1D).

Additionally, IL-23 did not affect IL-17A production in both co-cultures (Fig. 2A,E) while increasing IL-17F secretion only in PBMC/skin fibroblast (Fig. 2F). None of the cytokines nor the antibodies affected IL-6 secretion in a statistically significant manner (Fig. S2).

To resume this part, co-cultures between activated PBMC and stromal cells, from both synovium and skin, lead to the secretion of pro-inflammatory cytokines notably IL-17A and IL-17F with differential effects according to stromal cell origin.

### Effects of exogenous IL-17 cytokines or their inhibition on their receptor expression

Response to cytokines is mediated by membrane receptors usually made of two chains: IL-17RA/IL-17RC for IL-17R, IL-12β1/IL-12β2 for IL-12R and IL-12β1/IL-23R for IL-23R. The IL-17RA/IL-17RC complex binds IL-17A/IL-17A and IL-17F/IL-17F homodimers and IL-17A/IL-17F heterodimer.

**Figure 3:**
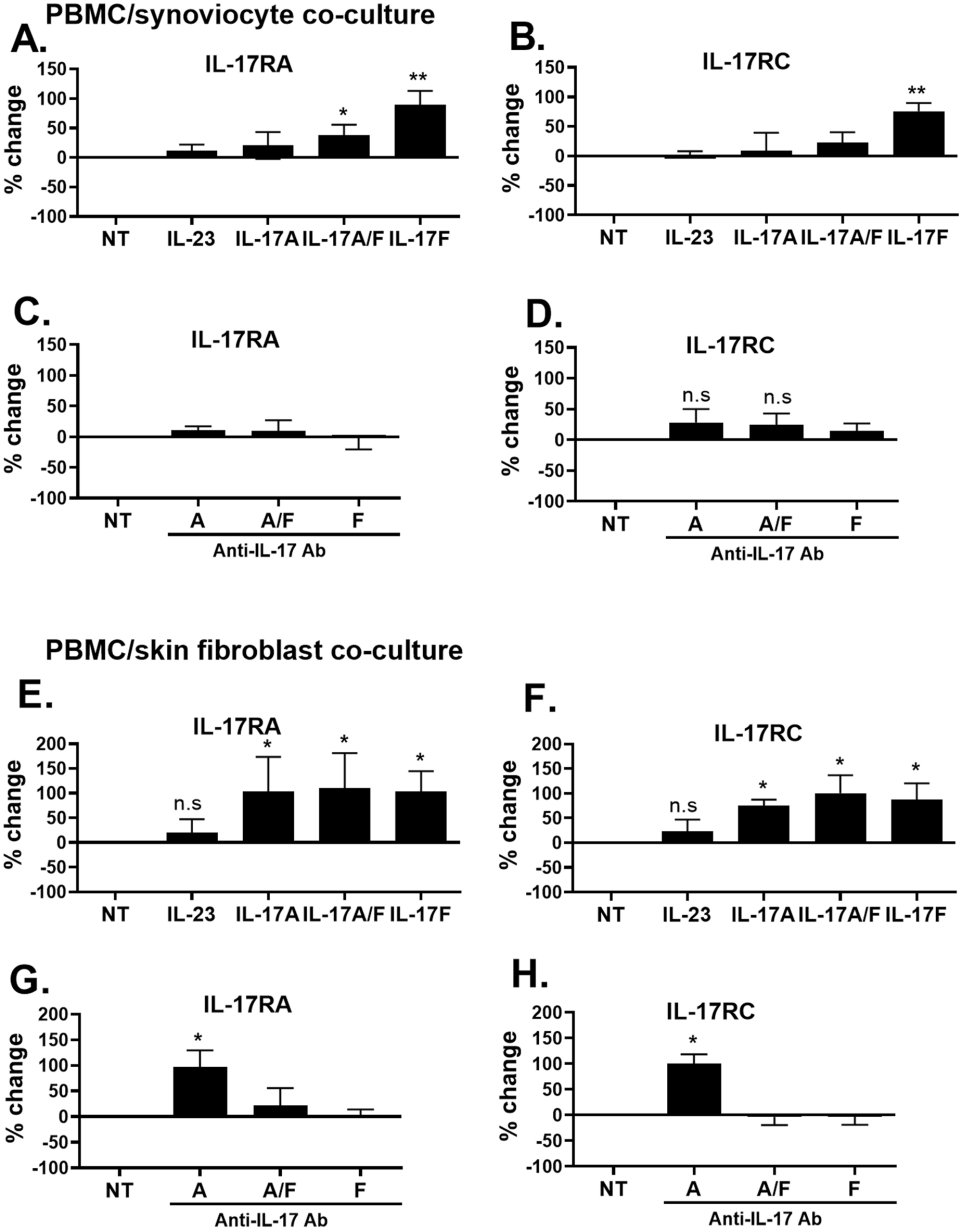
Effects of exogenous cytokines and antibodies on IL-17 receptor expression in co-cultures between PBMC and stromal cells from different origins. PHA-activated PBMC were treated or not with exogenous cytokines (IL-23, IL-17A, IL-17A/F and IL-17F) or antibodies (anti-IL-17A, anti-IL-17A/F and anti-IL-17F) and co-cultured with synoviocytes (**A–D**) or with skin fibroblasts (**E–H**) at a 5:1 ratio for 24 h. RNA was recovered and IL-17RA, IL-17RC, IL-23R, IL-12Rβ1 and IL-12Rβ2 expression was assessed by RT-QPCR. The results show the gene expression normalized by GAPDH expression. *p < 0.05; **p < 0.01. Receptor expression was expressed as a percentage of change compared to the control condition, which is the non-treated condition (NT) and used as 100% control. Results are presented as mean ± SD, n = 5 to 6 experiments.

In PHA-activated co-cultures of PBMC and skin fibroblasts or synoviocytes, mRNA expression of IL-17RA, IL-17RC, IL-23R, IL-12Rβ1 and IL-12Rβ2 was measured following 24 h of treatment with IL-23, IL-17A, IL-17A/F, IL-17F, anti-IL-17A, anti-IL-17A/F or anti-IL-17F antibodies. Results are presented in Fig. 3 and Supplemental Fig. S3. Receptor expression was expressed as a percentage of change compared to the control condition, which is the non-treated condition (NT) and used as 100% control.

In PBMC/synoviocyte co-cultures, IL-17F increased IL-17RA and RC (Fig. 3A,B), IL-23R and IL-12Rβ1 (Fig. S3A,B) expression as well as that of IL-12Rβ2 but not significantly (Fig. S3C). In contrast, IL-17A did not affect any receptor subunit expression (Fig. 3A,B, Fig. S3A–C) while IL-17A/F only increased IL-17R expression (IL-17RA: + 37.5%, p = 0.042, Fig. 3A and IL-17RC: + 22.6%, p = 0.075, Fig. 3B). This suggests that IL-17F but not IL-17A upregulated IL-17 receptor expression in PBMC/synoviocyte co-cultures.

Exogenous IL-23 and IL-17-targeting antibodies did not have any significant effect on IL-17R expression in PBMC/synoviocyte co-cultures (Fig. 3A–D). IL-12Rβ1 expression was decreased by anti-IL-17A/F and anti-IL-17F but not by anti-IL-17A (− 29.4%, − 28.1%, p = 0.0189 and p = 0.015 and − 14%, p = 0.087, respectively, Fig. S3E). IL-12Rβ2 was downregulated by anti-IL-17A and anti-IL-17A/F but not by anti-IL-17F antibodies (− 36%, − 39.5%, p = 0.0211 and p = 0.032, and − 16.3%, p = 0.078, respectively, Fig. S3F). IL-23R expression was not affected by anti-IL-17 antibodies (Fig. S3D).

In PBMC/skin fibroblast co-cultures, all IL-17 cytokines increased IL-17R mRNA expression (Fig. 3E,F). IL-17A increased both IL-23R and IL-12Rβ1 (Fig. S3G,H) while decreasing IL-12Rβ2 expression (Fig. S3I) thus favoring the expression of IL-23R and not IL-12R. On the other hand, IL-17A/F increased IL-23R and IL-12Rβ2 expression (Fig. S3G,I). IL-17F increased IL-12Rβ1 and IL-12Rβ2 (Fig. S3H,I), favoring the expression of IL-12R instead of that of IL-23R. Interestingly, IL-23 increased IL-23R expression (Fig. S3G) while decreasing IL-12Rβ2 expression (Fig. S3I) thus, favoring IL-23R but not IL-12R expression.

Anti-IL-17A antibody increased IL-17RA/RC expression and IL-23R (Fig. 3G,H, Fig. S3J). Moreover, anti-IL-17A decreased IL-12Rβ1 (Fig. S3K) and IL-12Rβ2 (Fig. S3L) thus IL-17A inhibition favored IL-17R and IL-23R over IL-12R expression. In addition, anti-IL-17A/F and anti-IL-17F decreased IL-23R, IL-12Rβ1 and IL-12Rβ2 (anti-IL-17A/F: − 29.9%; − 46.5%; − 33.6%; anti-IL-17F: − 24.1%; − 35.9%; − 37%, respectively, Fig. S3J–L).

To conclude this part, IL-17A and F had differential effects on their receptor subunit expression with skin fibroblast co-cultures being more sensitive to IL-17A and F than synoviocytes, where only IL-17F had a significant effect.

### Monocyte exclusion increases IL-17A and IL-17F levels in co-cultures

Considering the opposite roles of IL-6 and IL-10 on the Th17 pathway and the role of cell interactions in maintaining inflammation, and since monocytes are one of the main sources of IL-23, we investigated their potential contribution. Monocytes were partially removed by Ficoll–Percoll gradient separation (Fig. S4).

**Figure 4:**
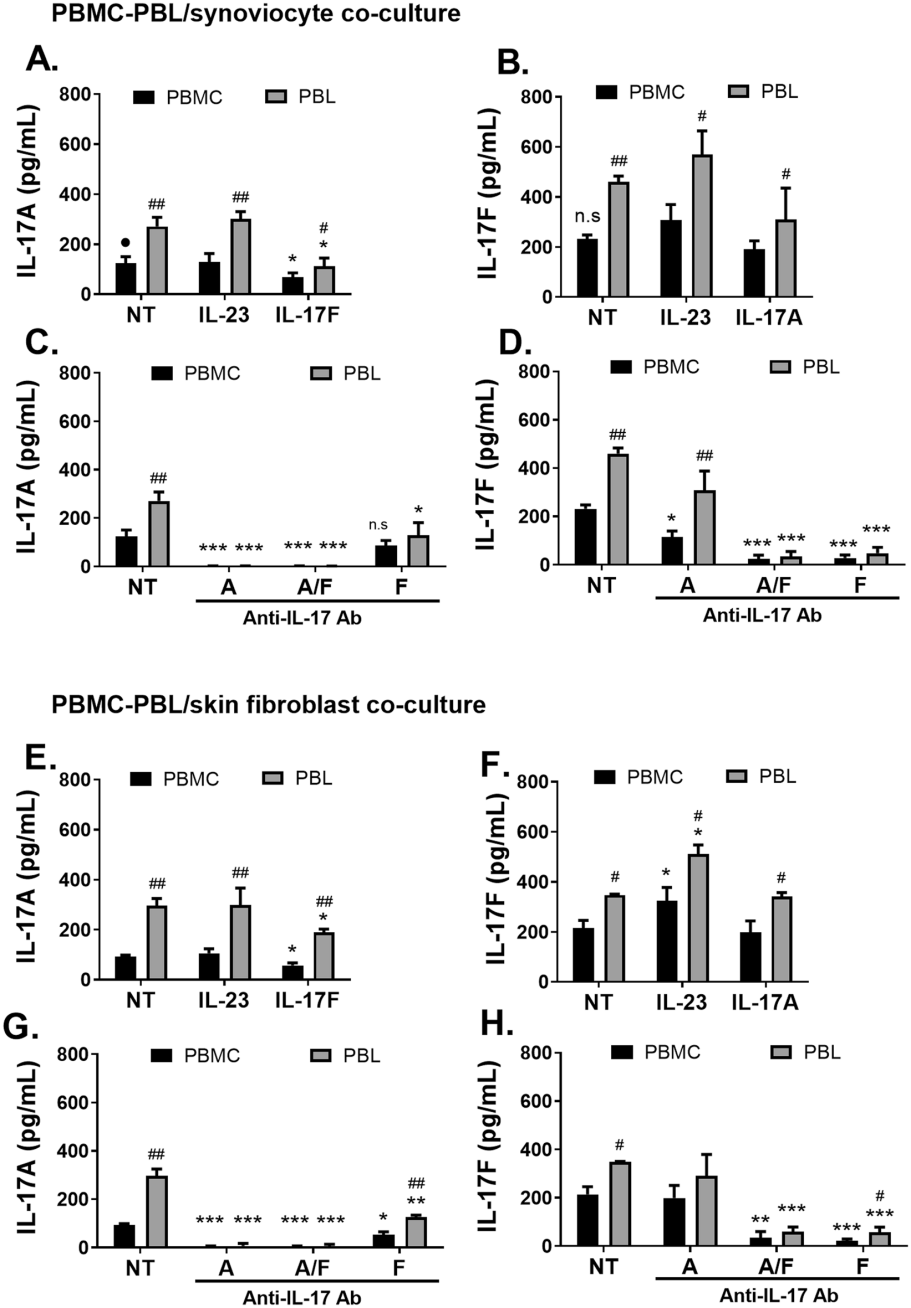
Effects of exogenous cytokines and antibodies on IL-17A and IL-17F production in co-cultures between PBMC or PBL and stromal cells from different origins. PHA-activated PBMC or PBL were treated or not with exogenous cytokines (IL-23, IL-17A, IL-17A/F and IL-17F) or antibodies (anti-IL-17A, anti-IL-17A/F and anti-IL-17F) and co-cultured with synoviocytes (**A–D**) or with skin fibroblasts (**E–H**) at a 5:1 ratio for 48 h. Production of IL-17A and IL-17F was measured by ELISA. “*” Compares the effect of the treatments or antibodies vs. the control condition. *p < 0.05; **p < 0.01; ***p < 0.005. “^#^” Compares the effect of monocyte exclusion on cytokine production (PBMC vs. PBL) within the same treatment condition. ^#^p < 0.05; ^##^p < 0.025. “•” Compares the effect of interaction with stromal cell from different origin on cytokine production (synoviocyte vs. skin fibroblast). ^ns^p > 0.05; ^•^p < 0.05. Results are represented as mean ± SD in pg/mL, n = 4 to 7 experiments.

PHA-activated peripheral blood lymphocytes (PBL) were co-cultured with synoviocytes or skin fibroblasts for 48 h, then, IL-17A, IL-17F, IL-6 and IL-10 production was assessed by ELISA. Results are presented in Fig. 4 and Supplemental Fig. S5.

IL-17A and IL-17F levels were upregulated in PBL/synoviocyte co-cultures compared to PBMC/synoviocyte indicating that monocyte exclusion increased IL-17A and IL-17F production (Fig. 4A,B). IL-17F decreased IL-17A secretion in PBL/synoviocyte co-cultures (Fig. 4A) as seen with PBMC co-cultures. Anti-IL-17A and anti-IL-17A/F decreased IL-17A levels in PBL/synoviocyte co-cultures while anti-IL-17F mediated a weaker downregulation (1.1 ± 0.8, 0.6 ± 0.06 and 128.8 ± 30.2 vs. 270.7 ± 21.3 pg/mL respectively, Fig. 4C). IL-17F production was not affected by IL-17A while both anti-IL-17A/F and anti-IL-17F antibodies strongly decreased IL-17F levels (310.2 ± 62.7, NS, 34.5 ± 10.3, 48.2 ± 11.8 vs. 459.8 ± 11.8 pg/mL, respectively, Fig. 4B,D).

Additionally, monocyte exclusion decreased IL-6 levels in PBL/synoviocyte co-cultures compared to PBMC (Fig. S5A). IL-6 production was not affected by cytokines in PBL/synoviocyte co-cultures (Fig. S5A). Conversely, all antibodies decreased IL-6 levels in PBL/synoviocyte co-cultures (Fig. S5C).

IL-10 is an important anti-inflammatory cytokine, mostly secreted by regulatory T cells (Treg) and monocytes^17^ and is implicated in the pathophysiology of inflammatory diseases via the suppression of IL-17^18^. IL-10 production was significantly reduced in PBL vs. PBMC/synoviocyte co-cultures (Fig. S5B). Overall, IL-10 production, from PBL/synoviocyte co-cultures, was not affected by cytokines, as in PBMC (Fig. S5B). Anti-IL-17A/F and anti-IL-17F antibodies decreased IL-10 levels (151.5 ± 13.4, 136.6 ± 21.9, vs. 253.8 ± 7.9 pg/mL, respectively, Fig. S5D). As in PBMC, IL-23 addition had no significant effect on cytokine secretion in PBL/synoviocyte co-cultures (Fig. 4A,B, Fig. S5A,B).

In PBL/skin fibroblast co-cultures, IL-17A and IL-17F were also upregulated compared to PBMC/skin fibroblast co-cultures indicating that monocyte exclusion, increased IL-17A and IL-17F production (Fig. 4E,F). IL-17A levels upregulation without monocytes was higher in PBL/skin fibroblast compared to PBL/synoviocyte co-cultures (threefold vs. twofold increase, p = 0.023, Fig. 4A,E) whereas IL-17F production increase was slightly lower in PBL/skin fibroblast co-cultures (1.6-fold vs. twofold increase, Fig. 4B,F). IL-17F decreased IL-17A secretion in PBL/skin fibroblast co-cultures (Fig. 4E) with no effect of IL-17A over IL-17F production (Fig. 4F), like PBMC/skin fibroblast co-cultures. Anti-IL-17A and anti-IL-17A/F decreased IL-17A levels in PBL/skin fibroblast co-cultures while anti-IL-17F mediated a weaker downregulation (6.9 ± 5.9, 6.2 ± 4.3, and 126.7 ± 4.4 vs. 295.6 ± 16.8 pg/mL, respectively, Fig. 4G). Conversely to PBL/synoviocyte co-cultures, IL-23 increased IL-17F production in PBL/skin fibroblast co-cultures (340.8 ± 9.4 and 511.1 ± 21.1 vs. 347.8 ± 1.6 pg/mL, respectively, Fig. 4F). Anti-IL-17A/F and anti-IL-17F strongly decreased IL-17F levels (Fig. 4H).

Monocyte exclusion decreased IL-6 production by twofold in PBL/skin fibroblast co-cultures compared to PBMC’s (Fig. S5E). IL-6 levels were nearly twofold higher in both PBMC and PBL/skin fibroblast co-cultures compared with PBMC and PBL/synoviocyte co-cultures (PBMC: 214.3 ± 5.9 vs. 114.2 ± 3.2 ng/mL; PBL: 100.1 ± 3.8 vs. 56 ± 3.5 ng/mL, respectively, Fig. S5A,E). IL-17A increased IL-6 secretion while IL-17F decreased it, in PBL/skin fibroblast co-cultures (Fig. S5E). Anti-IL-17A, anti-IL-17A/F and anti-IL-17F decreased IL-6 secretion in PBL/skin fibroblast co-cultures (19 ± 5.8, 19 ± 9.3 and 38.2 ± 7.1 vs. 100.1 ± 3.8 ng/mL, respectively, Fig. S5G) while lesser effect of the antibodies on IL-6 secretion was seen in PBMC/synoviocyte co-cultures (Fig. S5G).

IL-10 production was twofold lower in PBL/skin fibroblast co-cultures compared to PBMC’s (Fig. S5F). IL-10 production was decreased by IL-17 cytokines in PBL/skin fibroblast co-cultures (IL-17A: 98.5 ± 4.2, IL-17A/F: 106.9 ± 10.8 and IL-17F: 96.7 ± 13.1 vs. 162.4 ± 12.2 pg/mL, Fig. S5F) in contrast to PBMC’s (Fig. S5F). Additionally, IL-10 production was decreased by all antibodies (Fig. S5H) with PBMC/skin fibroblast co-cultures, however, in contrast, IL-10 production was not altered by any antibody in PBL/skin fibroblast co-cultures (Fig. S5H). As in PBMC, IL-23 addition had no significant effect on IL-10 secretion in PBL/skin fibroblast co-cultures (Fig. S5F). IL-10 was not directly involved in IL-17A and IL-17F regulation as exogenous IL-10 or anti-IL-10 antibody did not affect IL-17A and F secretion in our co-culture models in both synoviocytes and skin fibroblasts (data not shown).

To resume, partial monocyte exclusion led to a decrease in IL-10 and IL-6 levels and a significant increase in IL-17A and F levels.

## Discussion

**Figure 5:**
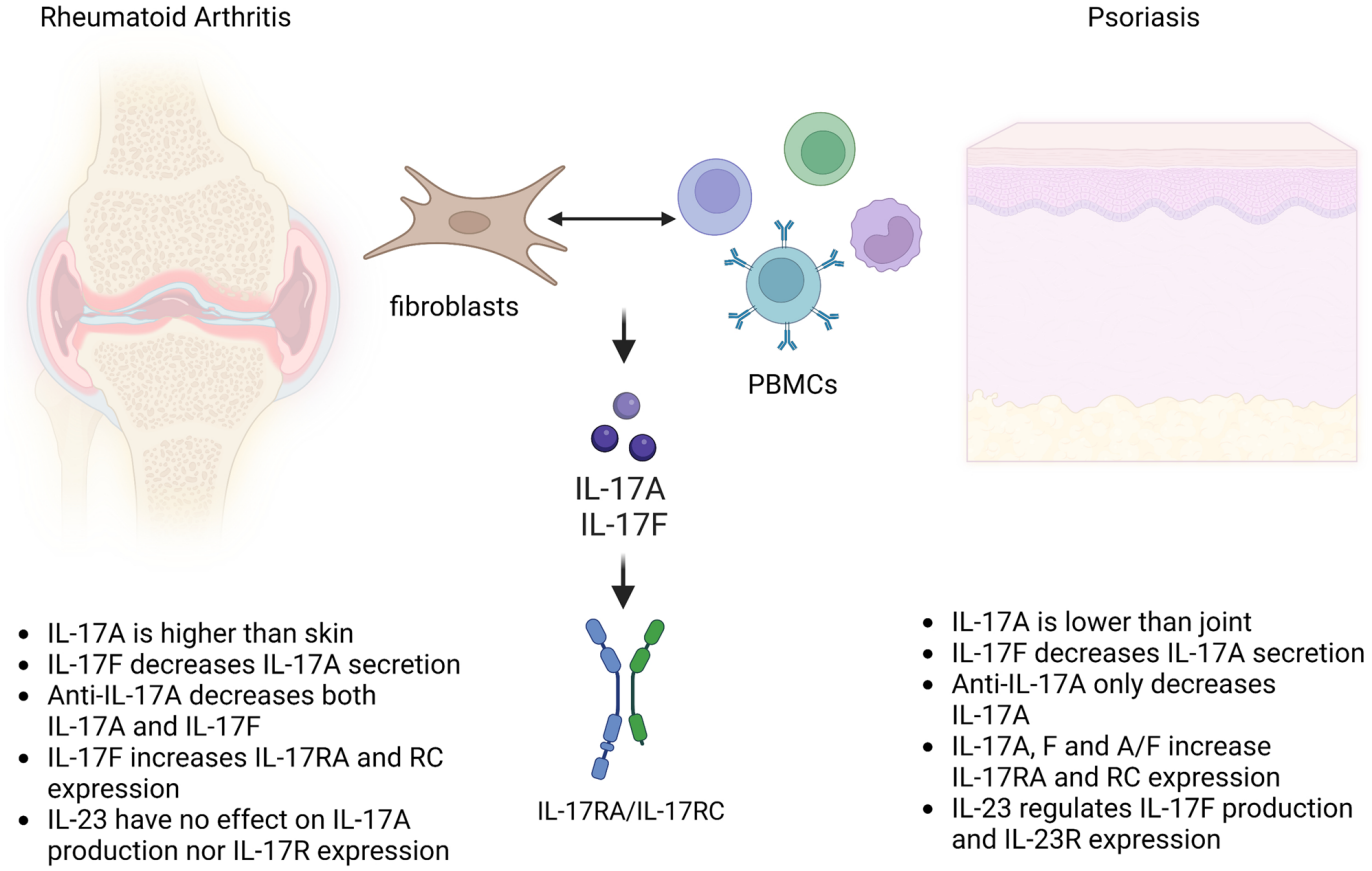
Differential contribution of IL-17A and IL-17F in co-cultures between PBMC and stromal cells from joint vs. skin. Co-cultures between PBMC and both synoviocytes from rheumatoid arthritis patients and skin fibroblasts from Psoriasis patients, lead to IL-17A and IL-17F which then bind to their receptor (IL-17RA/IL-17RC) to mediate their effects. Main differences of IL-17A and IL-17F contributions between skin and joint are listed.

IL-17A has emerged as pivotal in driving tissue pathology in immune-mediated inflammatory diseases and has been the main target of biologic medications. The role of IL-17F, sharing 55% sequence homology and overlapping biological function, remains less clear. Here, we evaluated the effects of IL-17A and IL-17F on interactions between immune cells and stromal cells from two clinical extremes: Pso vs. RA, with Pso showing high sensitivity to IL-17/IL-23 inhibition and RA with poor or no sensitivity. Our results show a key differential effect of IL-17 cytokines according to stromal cell origin (Fig. 5).

First, cell interactions alone were sufficient to induce IL-6 secretion which was higher in skin fibroblast vs. synoviocyte co-cultures. A high IL-17A secretion necessitated both activation and cell interactions and was notably higher in synoviocyte vs. skin fibroblast co-cultures. Conversely, PHA activation alone was sufficient but further enhanced by cell interactions for IL-17F production. In both systems, IL-17F production was higher than that of IL-17A, consistent with findings in the literature: IL-17F expression was consistently higher (30-fold) in psoriatic lesional tissues and serum than IL-17A^19^. Other studies argue that this tendency is inversed in inflamed joint compared to skin^20^. Even if IL-17F effect is weaker than that of IL-17A, IL-17F local production could be higher because of the higher number of IL-17F-producing cells^21^. Inconsistencies across studies in levels of IL-17F detected in patient samples may be due to differences in the specific assay method, differences in sampling preparation or heterophilic antibody interference^22^.

In both skin and joint stromal cell co-cultures with PBMC, anti-IL-17A, anti-IL-17A/F and anti-IL-17F antibodies drastically reduced IL-17A and IL-17F secretion with some notable differences: anti-IL-17A and anti-IL-17A/F strongly decreased IL-17A while anti-IL-17F had a weaker effect in both models. IL-17F was strongly reduced using anti-IL-17A/F and anti-IL-17F antibodies. Anti-IL-17A, however, failed to mediate any effect on IL-17F in PBMC/skin fibroblast co-cultures while reducing, by only twofold, IL-17F in PBMC/synoviocyte co-cultures. These differences indicate a differential implication of IL-17A and IL-17F in both anatomic situations.

In both synoviocyte and skin fibroblast co-cultures, IL-17F decreased IL-17A secretion by twofold. Conversely, IL-17A did not significantly affect IL-17F secretion. Surprisingly, anti-IL-17F led also to a decrease in IL-17A production in co-cultures. The effect of IL-17F on IL-17A production and vice versa is currently being investigated and can help decipher the precise implication of both cytokines in RA and Pso. Furthermore, IL-17A and IL-17F are differentially regulated upon T cell co-stimulation and have overlapping but also distinct functions in inflammatory diseases^23^. Only IL-17F upregulated IL-17R expression, in PBMC/synoviocyte co-cultures, while IL-17A, IL-17A/F and IL-17F mediated a similar effect, in PBMC/skin fibroblast co-cultures. This differential activity of IL-17A and IL-17F may be due to the competition of both cytokines for the same receptor, the IL-17RA-IL-17RC heterodimer and on their differential effects and regulation within local inflammation. In our model, blocking IL-17F may not be enough to restore IL-17R levels as there may be other factors involved in IL-17 receptor regulation. IL-17R regulation is still somewhat ambiguous in the literature and needs further investigation and currently, dynamic time-course experiments are being conducted in the team to tackle this. Moreover, IL-17RA and RC dimerization can affect the threshold for IL-17A and F signaling^24^.

In RA synoviocytes, IL-17A and IL-17F stimulated downstream signaling in a similar but not identical manner, with IL-17A regulating a much larger number of inflammation-related genes compared with IL-17F^25^. Indeed, IL-17 cytokines can act in an autocrine manner and can influence the function of other cytokines, including other IL-17 family members^26^. This may explain the activity of IL-17F on IL-17A secretion, suggesting a regulatory role for IL-17F. IL-17RA has a 100-fold weaker affinity for IL-17F than for IL-17A and an intermediate affinity for the IL-17A/F heterodimer. Conversely, IL-17RC has a higher affinity for IL-17F than for IL-17A^27^. Recent evidence suggests that these two cytokines play non-overlapping and even opposite roles^28^. It has been recently reported that a structure of IL-17F binds IL-17RC homodimers, while IL-17A does not, suggesting that IL-17F may trigger distinct IL-17RA-independent cellular functions, as compared to IL-17A^25, 29^. In RA synovial tissues, IL-17A, IL-17F and their receptors IL-17RA and RC were abundantly expressed with different expression patterns^30^. We show that blocking IL-23, IL-17A or F did not influence IL-17R in the case of PBMC/synoviocyte co-cultures while in PBMC/skin fibroblast co-cultures, anti-IL-17A increased IL-17R expression and decreased IL-23 receptor expression. Indeed, we saw, in our in vitro model, an effect on IL-17A blockade on IL-17R and IL-23R in Pso skin fibroblast but not in RA synoviocytes hinting to the well-established implication of the IL-23/IL-17 cytokines and their receptors in Pso. Targeting IL-17RA with brodalumab is highly effective and inhibits signaling induced by IL-17A, IL-17F and IL-17A/F^31, 32^ which is not the case yet for RA where no clinical efficacy is observed^33^. Whether varying receptor expression patterns^27^ coupled with the different affinity of each receptor chain for IL-17A or IL-17F underlies their diverging biological functions remains an open question.

Although it is traditionally accepted that IL-17A and IL-17F are produced primarily by CD4+ Th17 lymphocytes, many other cell types express them for instance, CD8+ T cells^34^, natural killer (NK) T cells^35^, ILC3^36^ and γ/δ T cells^37^, with the latter worth investigating since they do not require IL-23 for IL-17A and IL-17F production^38^.

Monocytes can recruit and promote the differentiation of T cells into inflammatory phenotypes in synovium^39^ and skin^40^. Here, we show that partial monocyte depletion decreased IL-10 and IL-6 in both co-cultures, confirming the role of monocytes as main producers of IL-10. Consequently, the depletion upregulated both IL-17A and IL-17F induced by cell interactions between PBMC and both stromal cells. IL-17A and IL-17F were not directly regulated by IL-10, which may indicate a selective IL-10 contribution according to the cell origin and the pathological context. Monocyte depletion has been shown to reduce IL-10 production and resulted in an increased IFNγ^17^. These results are somewhat contradictory with other studies that show an IL-17A activating role for monocytes through IL-6 and IL-1β^41^. This tendency of upregulated IL-17A and F was reversed in our older studies as monocyte exclusion led to a decrease of IL-17A in the case of skin fibroblast^14^ with no significant effect in synoviocytes^13^. This can be due to the monocyte depleting method used in those studies which was by adherence and only led to the exclusion of ≈ 50% of monocytes among PBMC while the double density Ficoll–Percoll used in the current study is more efficient and depletes ≈ 70–90% of monocytes. T cell activation method may as well influence IL-10 secretion by monocyte in RA^42^. Although the functions of monocytes and T cells have been investigated for many years, the study of their interactions in RA has been scarcely approached and needs further investigation.

In line with the results described here, dual blockade of IL-17A and F (Bimekizumab) has been shown to be more effective than blockade of IL-17A alone in Pso^43^. A recent phase IIa proof-of-concept study assessed dual blockade of IL-17A and IL-17F in RA patients with inadequate TNF-α response. A greater reduction was seen in DAS28-CRP in the anti-TNF inadequate response plus bimekizumab group compared to anti-TNF inadequate response plus placebo group^44^. Several hypotheses emerge as to why IL-17A blockade is not as successful in RA as could reasonably be expected from experimental and in vitro models. Upon blocking IL-17A alone, the synergistic effects of IL-17F with TNF can still induce potent inflammatory effects^21^. The heterogeneous and somewhat conflicting expression pattern of IL-17A and IL-17F^20, 45^, as well as the differential detection of circulating bioactive IL-17A^46, 47^ may also reflect the limited clinical response.

In conclusion, these results denote key differences between IL-17A and IL-17F in their regulation, their mediated effects on immune cell/stromal cell co-cultures and thus their differential implication according to stromal cell origin. Skin fibroblasts appear more sensitive than synoviocytes. The understanding of such differences could provide, at least in part, an explanation for some of the unexpected differences in response to IL-17 inhibitors depending on diseases.

## Materials and methods

Synoviocytes were obtained from synovial tissue of RA patients undergoing joint surgery and who fulfilled the American College of Rheumatology criteria for RA^48^. Skin fibroblasts were obtained from skin biopsies of psoriatic patients who fulfilled the Classification Criteria for Pso^14^. Synovial and skin biopsies were minced then adhered in 6-well plates in Dulbecco’s modified Eagle’s medium (DMEM; Eurobio, Courtaboeuf, France) supplemented with 10% fetal bovine serum (FBS; Life Technologies, Carlsbad, USA), 2 mM l-glutamine and 100 U/mL penicillin/streptomycin. Cells were maintained at 37 °C in a humidified 5% CO2 incubator and used between passages 4 and 9. PBMC from healthy donors were isolated by Ficoll-Hypaque (Eurobio, Courtaboeuf, France) density-gradient centrifugation. Everyone signed an informed consent form. The protocol was approved by the Ethics Committee of the Hospitals of Lyon (AC-2016-272) and complies with the Helsinki Declaration.

### Co-culture assays

Co-culture was initiated by seeding synoviocytes or skin fibroblasts overnight in 96-well or 24-well plates at a density of 2 × 10^4^ cells or 1 × 10^5^ cells/well, respectively in RPMI 1640 medium (Eurobio, Courtaboeuf, France) supplemented with 10% FBS, 2 mM l-glutamine, and 100 U/mL penicillin/streptomycin (complete RPMI). The next day, healthy PBMC (1 × 10^5^ cells or 5 × 10^5^ cells/well) were pre-incubated in complete RPMI with or without treatments and then seeded at a 5:1 ratio, in the presence of phytohemagglutinin (PHA, 5 μg/mL). After 24 h, cell mRNA was collected for analysis of receptor expression and after 48 h, supernatants were collected for analysis of cytokine secretion. Co-cultures were done with 5 different cell lines issued from five patients for synoviocytes and for skin fibroblasts. Cell passage as well as cell densities per well and experiment set-up was similar between synoviocyte and skin fibroblast co-cultures. Both cell lines were set each time in parallel when comparing skin vs. synovium.

### Treatments

Treatments evaluated in co-cultures consisted of exogenous cytokines: IL-17A, IL-17A/F, IL-17F and IL-23, or antibodies directed against IL-17 family members: anti-IL-17A (Secukinumab), anti-IL-17A/F (Bimekizumab) and anti-IL-17F (kindly provided by Dr Ash Maroof, UCB Slough, UK). Concentrations used in co-cultures were 50 ng/mL for cytokines and 1 µg/mL for antibodies. A control antibody, IgG1 directed against the BetV1 allergen was used at the same concentration (Dendritics, Lyon, France).

### Enzyme-linked immunosorbent assays (ELISA)

IL-17A, IL-17F, IL-6 and IL-10 levels in culture supernatants were measured with Diaclone ELISA kits (Diaclone, Besançon, France).

### RNA extraction and real-time PCR

Total RNA was extracted from both PHA-activated PBMC and corresponding stromal cells (Pso skin fibroblasts or RA synoviocytes) using the RNeasy Mini Kit (Qiagen®, Hilden, Germany) and quantified with the Quant-it kit assay (Invitrogen™ by Thermo Fisher Scientific, Grand Island, NY, USA). cDNA was synthesized using the QuantiTect reverse transcription kit (Qiagen®) according to the manufacturer’s instructions. SYBR green-based real-time qRT-PCRs were performed on the CFX96 Real-Time PCR Detection System (BioRad, Hercules, CA, USA) using the QuantiFast SYBR green kit and QuantiTect primers (Qiagen®). Cycle threshold values were normalized with respect to the endogenous control gene glyceraldehyde 3-phosphate dehydrogenase (GAPDH). The relative expression of tested genes in the different conditions was determined using the comparative threshold cycle method as described by the manufacturer.

### Monocyte contribution

Monocytes from healthy donors were isolated from human peripheral blood by density gradient centrifugation using Uni-sep maxi tubes (Eurobio scientific, Les Ulis, France) at 400×*g* for 20 min and then by centrifugation on a 50% Percoll solution at 400×*g* for 20 min. Cells were then stained for 20 min at 4 °C in staining buffer (PBS 1× + 2% of FBS) with CD14-FITC (Thermo Fisher Scientific, eBiosciences), CD3-EFluor450 (Thermo Fisher Scientific, Invitrogen), CD4-PE-Cy7 (Thermo Fisher Scientific, Invitrogen) and CD19-PB (Thermo Fisher Scientific, Invitrogen), washed, and analyzed by flow cytometry (Navios, Beckman Coulter, Brea, CA, USA). Analysis was done with the FlowJo software. Recovered monocytes were around 80–90% pure as assessed by CD14 flow cytometry labeling.

### Statistical analysis

Statistical analyses were performed using either a paired nonparametric Wilcoxon test or a non-paired Mann–Whitney test when comparing the effect of treatments on samples from the same stromal origin. When different cell co-cultures and different cytokines were compared, a nonparametric Kruskal–Wallis test followed by a posthoc Dunn’s test were used. All analyses were performed with Graph Pad Prism 6 software. p values < 0.05 were considered as significant.

### Ethical approval information

The protocol was approved by the Ethics Committee of the Hospitals of Lyon under the number AC-2016-272.

### Supplementary Information

Supplementary Figures.

## Data Availability

All data relevant to the study are included in the article.
