# Supplementary material for: Differential effects of interleukin-17A and 17F on cell interactions between immune cells and stromal cells from synovium or skin
Source: Sci Rep. 2023 Nov 6;13:19223. doi: 10.1038/s41598-023-45653-8 (PMC10628108; doi:10.1038/s41598-023-45653-8)
Supplement: Supplementary file 1 — Supplementary Figures. [file 41598_2023_45653_MOESM1_ESM.pdf]

Differential effects of Interleukin-17A and 17F on cell interactions between immune cells and stromal cells from synovium or skin

Issam Tout[1], Mélissa Noack[1], Pierre Miossec[1,2]

[1] Immunogenomics and Inflammation research Unit, Edouard Herriot Hospital, Hospices Civils de Lyon, Lyon, France

[2] Department of Clinical Immunology and Rheumatology, Edouard Herriot Hospital, 5 place d'Arsonval, 69437 Lyon, France

*Corresponding author: Pr. Pierre Miossec, Immunogenomics and Inflammation research Unit, Edouard Herriot Hospital, 5 Place d'Arsonval, 69003, Lyon, France   Email: pierre.miossec@univ-lyon1.fr

Supplemental figures

Fig. S1

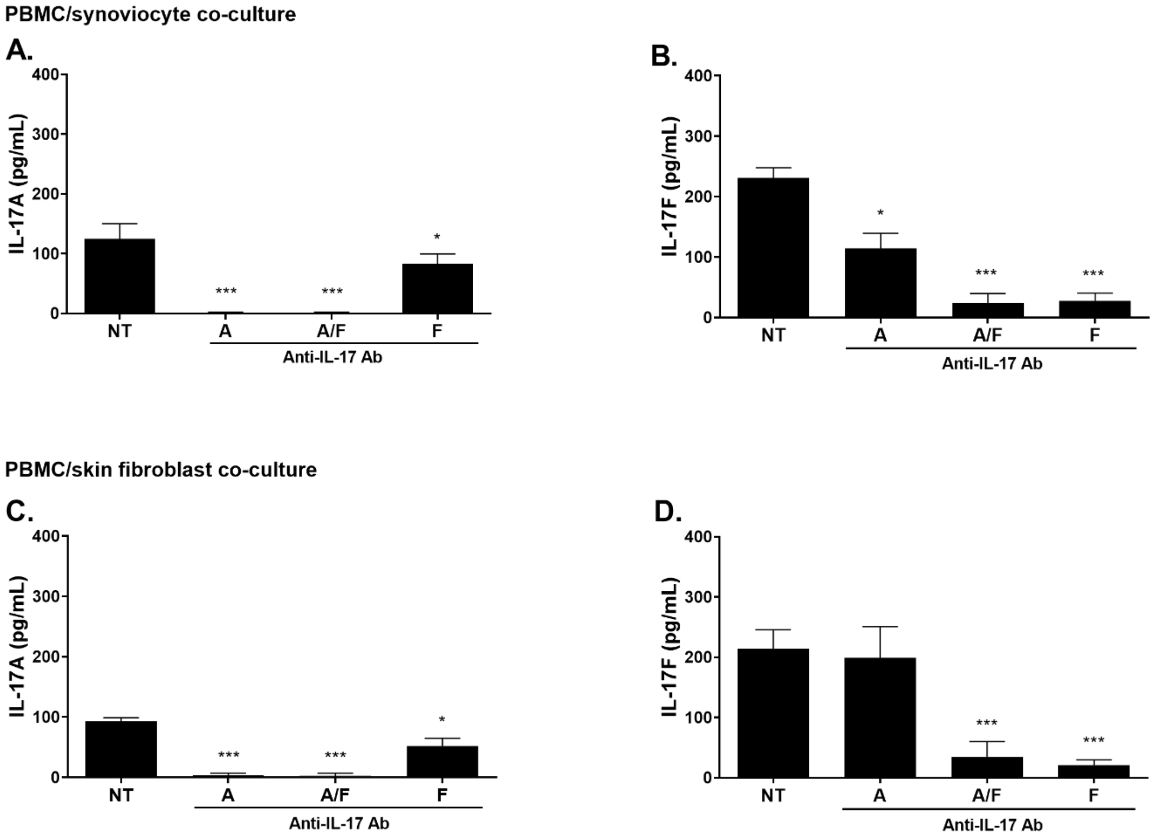

Fig. S2

PBMC/synoviocyte co-culture

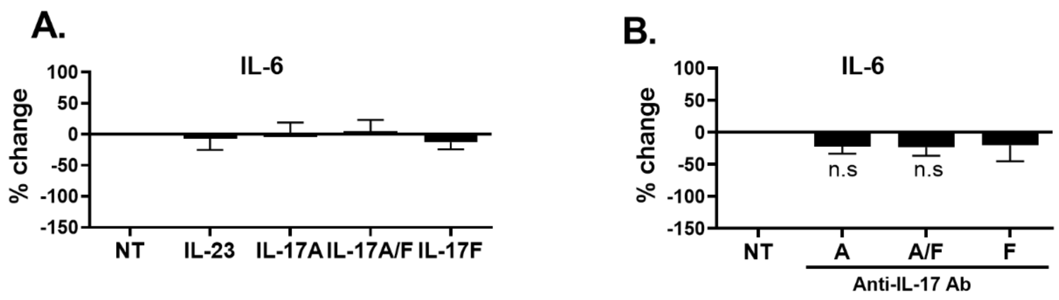

PBMC/skin fibroblast co-culture

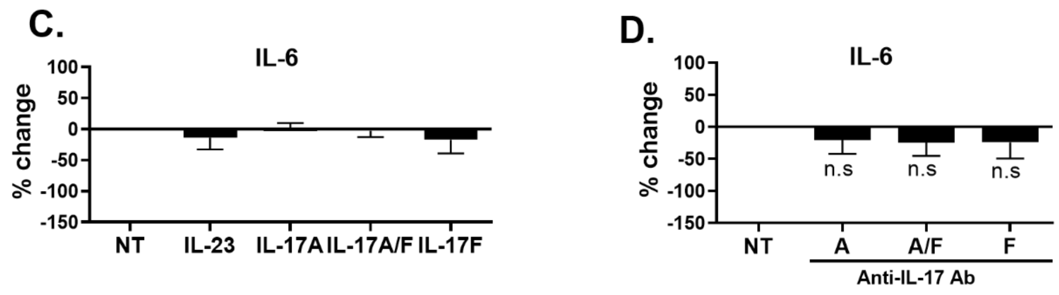

Fig. S3

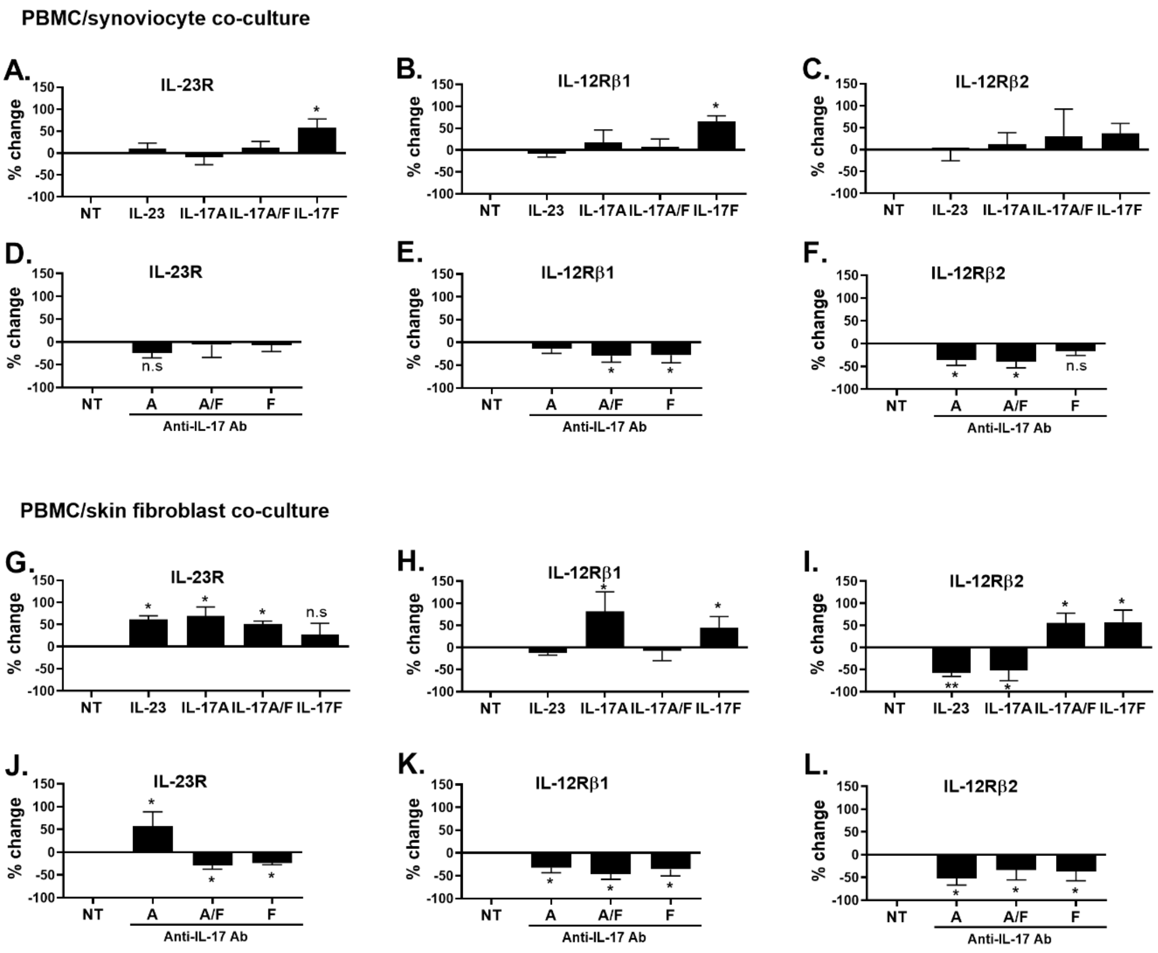

PBMC/synoviocyte co-culture
A.
IL-23R
B.
IL-12Rβ1
C.
IL-12Rβ2
% change
150
100
50
0
-50
-100
NT
IL-23
IL-17A
IL-17A/F
IL-17F
*
D.
IL-23R
E.
IL-12Rβ1
F.
IL-12Rβ2
A
A/F
F
Anti-IL-17 Ab
n.s
PBMC/skin fibroblast co-culture
G.
IL-23R
H.
IL-12Rβ1
I.
IL-12Rβ2
**
J.
IL-23R
K.
IL-12Rβ1
L.
IL-12Rβ2

Fig. S4

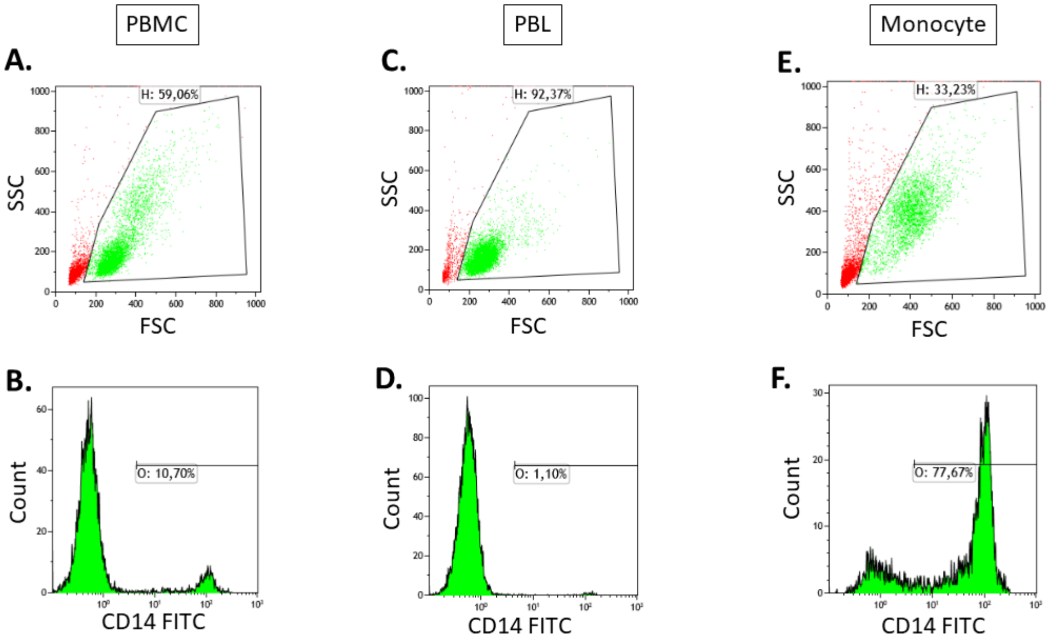
PBMC
PBL
Monocyte
A.
H: 59,06%
SSC
FSC
C.
H: 92,37%
SSC
FSC
E.
H: 33,23%
SSC
FSC
B.
O: 10,70%
Count
CD14 FITC
D.
O: 1,10%
Count
CD14 FITC
F.
O: 77,67%
Count
CD14 FITC

Fig. S5

PBMC-PBL/synoviocyte co-culture

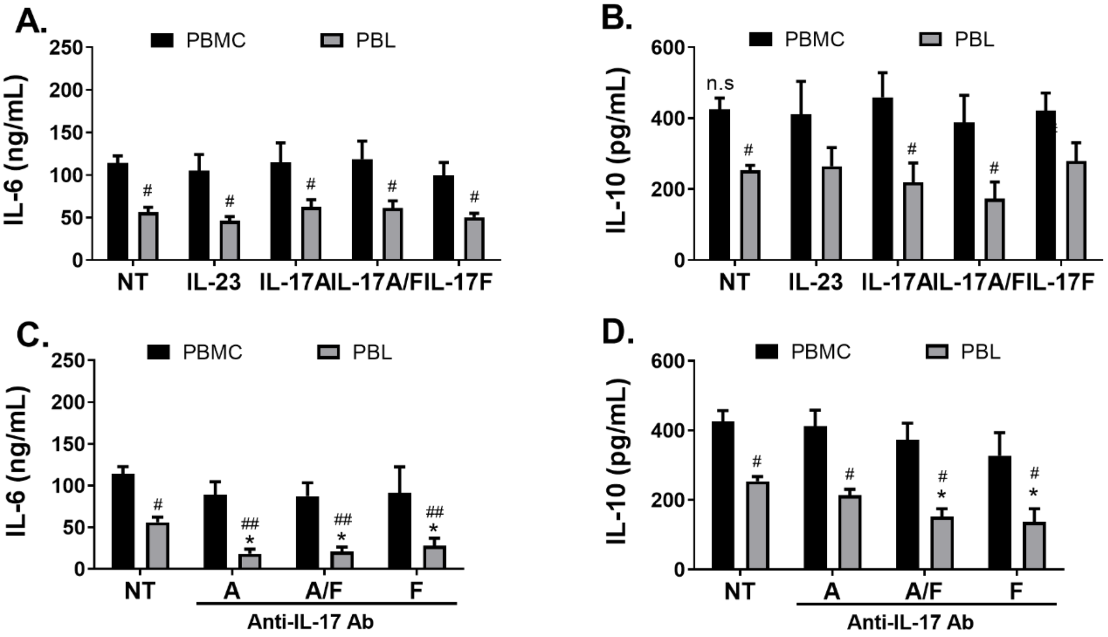

PBMC-PBL/skin fibroblast co-culture

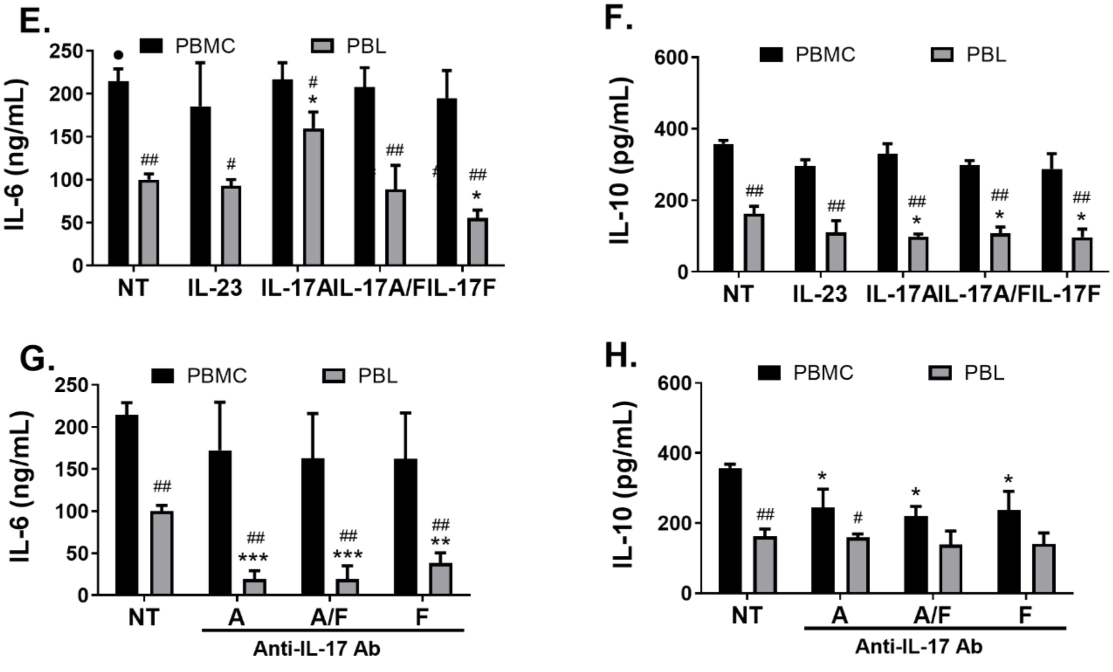

Supplemental Figure legends

Figure S1. ***Effects of anti-IL-17 antibodies on IL-17A and IL-17F production in co-cultures between PBMC and stromal cells from different origins.***

PHA-activated PBMC were treated or not with anti-IL-17 antibodies (anti-IL-17A, anti-IL-17A/F and anti-IL-17F) and co-cultured with synoviocytes (A, B) or with skin fibroblasts (C, D) at a 5:1 ratio for 48h. Production of IL-17A and IL-17F was measured by ELISA. *p<0.05; **p< 0.01; ***p< 0.005. Results are presented in pg/mL, as mean ± SD, n = 5 to 7 experiments.

Figure S2. ***Effects of exogenous cytokines and anti-IL-17 antibodies on IL-6 production in co-cultures between PBMC and stromal cells from different origins.***

PHA-activated PBMC were treated or not with exogenous cytokines (IL-23, IL-17A, IL-17A/F and IL-17F) or antibodies (anti-IL-17A, anti-IL-17A/F and anti-IL-17F) and co-cultured with synoviocytes (A, B) or with skin fibroblasts (C, D) at a 5:1 ratio for 48h. Production of IL-6 was measured by ELISA. *p<0.05; **p< 0.01; ***p< 0.005. Cytokine production was expressed as a percentage of change compared to the control condition, which is the non-treated condition (NT) and used as 100% control. Results are presented as mean ± SD, n = 5 to 7 experiments.

Figure S3. ***Effects of exogenous cytokines and antibodies on IL-23/IL-12 receptor subunits expression in co-cultures between PBMC and stromal cells from different origins.***

PHA-activated PBMC were treated or not with exogenous cytokines (IL-23, IL-17A, IL-17A/F and IL-17F) or antibodies (anti-IL-17A, anti-IL-17A/F and anti-IL-17F) and co-cultured with synoviocytes (A, B, C, D, E, F) or with skin fibroblasts (G, H, I, J, K, L) at a 5:1 ratio for 24h. RNA was recovered and IL-23, IL-12Rβ1 and IL-12Rβ2 expression was assessed by RT-QPCR. The results show the gene expression normalized by GAPDH expression. $^{*}p<0.05$; $^{**}p< 0.01$. Receptor expression was expressed as a percentage of change compared to the control condition, which is the non-treated condition (NT) and used as 100% control. Results are presented as mean ± SD, n = 5 to 6 experiments.

Figure S4. ***Gating strategy for flow cytometry analysis of monocyte depletion.***

PBMC and subsequently PBLs and monocytes were obtained by successive Ficoll and Percoll gradient separation. Cells were then stained for 20 min at 4°C in staining buffer (PBS 1X + 2% of FBS) with CD14-FITC, CD3-EFluor450, CD4-PE-Cy7 and CD19-PB, washed, and analyzed by flow cytometry. Analysis was done with the FlowJo software. Representative plots and histograms of PBMC (A, B), PBLs (C, D) and monocytes (E, F) are shown. Monocytes were 70-90% depleted in the PBL fraction.

Figure S5. ***Effects of exogenous cytokines and antibodies on IL-6 and IL-10 production in co-cultures between PBMC or PBL and stromal cells from different origins.***

PHA-activated PBMC or PBL were treated or not with exogenous cytokines (IL-23, IL-17A, IL-17A/F and IL-17F) or antibodies (anti-IL-17A, anti-IL-17A/F and anti-IL-17F) and co-cultured with synoviocytes (A, B, C, D) or with skin fibroblasts (E, F, G, H) at a 5:1 ratio

for 48h. Production of IL-6 and IL-10 was measured by ELISA. “*” compares the effect of the treatments or antibodies vs. the control condition. *$p<0.05$; **$p< 0.01$; ***$p<0.005$. “#” compares the effect of monocyte exclusion on cytokine production (PBMC vs. PBL) within the same treatment condition. $^{\#}p < 0.05$; $^{\#\#}p< 0.025$. “•” compares the effect of interaction with stromal cell from different origin on cytokine production (synoviocyte vs. skin fibroblast). n.s $p>0.05$; • $p<0.05$. Results are represented in ng/mL for IL-6 and in pg/mL for IL-10, as mean ± SD, n = 4 to 7 experiments.
